# Calmodulin Binding to Dfi1p Promotes Invasiveness of *Candida albicans*

**DOI:** 10.1371/journal.pone.0076239

**Published:** 2013-10-14

**Authors:** Talya R. Davis, Paola C. Zucchi, Carol A. Kumamoto

**Affiliations:** 1 Program in Molecular Microbiology, Sackler School of Graduate Biomedical Sciences, Tufts University, Boston, Massachusetts, United States of America; 2 Department of Molecular Biology and Microbiology, Tufts University School of Medicine, Boston, Massachusetts, United States of America; New Jersey Medical School, Rutgers University, United States of America

## Abstract

*Candida albicans*, a dimorphic fungus, undergoes hyphal development in response to many different environmental cues, including growth in contact with a semi-solid matrix. *C. albicans* forms hyphae that invade agar when cells are embedded in or grown on the surface of agar, and the integral membrane protein Dfi1p is required for this activity. In addition, Dfi1p is required for full activation of mitogen activated protein kinase Cek1p during growth on agar. In this study, we identified a putative calmodulin binding motif in the C-terminal tail of Dfi1p. This region of Dfi1p bound to calmodulin *in vitro*, and mutations that affected this region affected both calmodulin binding *in vitro* and invasive filamentation when incorporated into the full length Dfi1p protein. Moreover, increasing intracellular calcium levels led to calcium-dependent, Dfi1p-dependent Cek1p activation. We propose that conformational changes in Dfi1p in response to environmental conditions encountered during growth allow the protein to bind calmodulin and initiate a signaling cascade that activates Cek1p.

## Introduction

Invasive candidiasis is the fourth most common nosocomial bloodstream infection in the United States. The dimorphic fungus *C. albicans* is responsible for the vast majority of these cases [1]. *C. albicans* can switch from a yeast to filamentous morphology in response to a wide variety of environmental conditions, including growth in contact with an agar matrix [2], and these changes in morphology are important for *C. albicans* pathogenesis (reviewed in [3], [4], [5], [6]).

Several *C. albicans* signaling pathways are involved in sensing environmental cues and promoting filamentation. The mitogen activated protein kinase (MAPK) Cek1p plays an important role in hyphae development on solid media (reviewed in [7]). The protein kinase A pathway is a second pathway that also regulates hyphae development (reviewed in [8]).

When cells are grown in contact with agar, either by embedding the cells within the agar matrix or by culturing cells on the surface of medium, filamentous growth of cells within the agar is observed [2]. Two different MAPKs, Cek1p and Mkc1p, are activated when cells are growing in contact with agar [2], [9], [10], [11]. Activation of Cek1p under these conditions is partially dependent on Dfi1p, an integral membrane protein that is important for filamentation in response to growth in contact with an agar matrix [12]. Dfi1p is also important for growth of *C. albicans* in the presence of cell wall targeting agents such as caspofungin or Congo red [12].

To understand the mechanism by which growth in contact with agar activated Dfi1p-dependent Cek1p activation, the sequence of the Dfi1p protein was analyzed. The C-terminal tail was found to contain a putative calmodulin binding motif, raising the possibility that Dfi1p binds calmodulin. Calmodulin, a ubiquitous eukaryotic protein involved in sensing and responding to calcium levels, is involved in filamentation in both *C. albicans* and the model yeast *Saccharomyces cerevisiae* [13], [14], [15], [16]. Despite this similarity in function of *C. albicans* and *S. cerevisiae* calmodulin, *C. albicans* calmodulin shares more sequence homology with mammalian calmodulin than with *S. cerevisiae* calmodulin and contains four calcium binding sites [17].

The goal of this study was to demonstrate a connection between Dfi1p and calmodulin and to understand the role this connection plays in the functions of Dfi1p. We show that the C-terminal tail of Dfi1p binds calmodulin. Furthermore, we demonstrate that mutations that disrupt the calmodulin binding domain of Dfi1p affect filamentation and MAPK activation in response to contact with an agar matrix and in response to increased intracellular calcium levels. We propose that during signaling, Dfi1p binds at least transiently to calmodulin; binding to calmodulin then allows Dfi1p to initiate a signaling cascade that activates Cek1p.

## Results

### Binding of calmodulin to the cytoplasmic tail of Dfi1p *in vitro*

**Figure 1:**
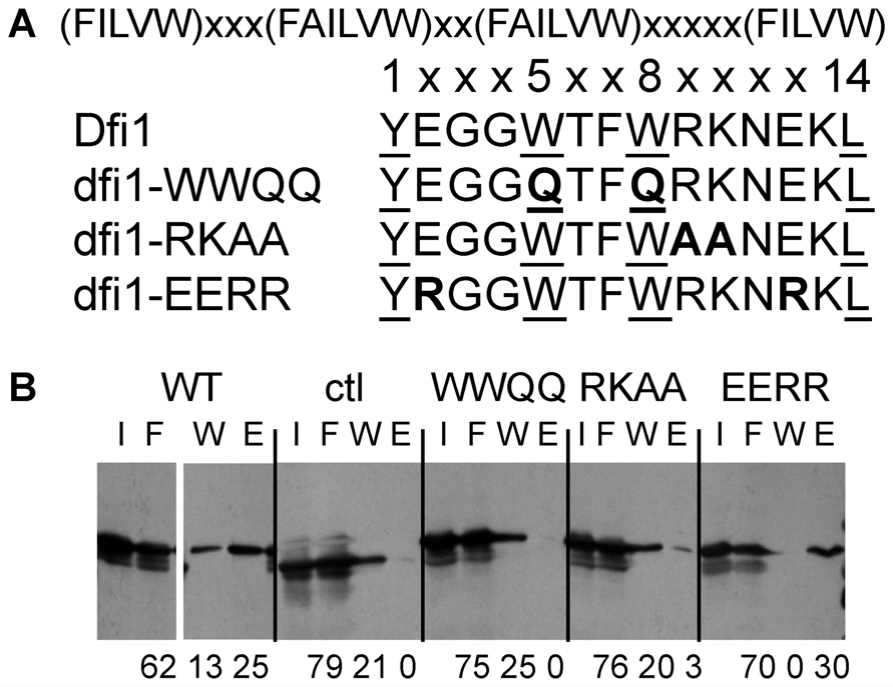
The C-terminal tail of Dfi1p binds to calmodulin *in vitro*. A, The consensus sequence of the 1-5-8-14 calmodulin binding motif is shown along the top. The wild type Dfi1p calmodulin binding region is shown. Hydrophobic residues corresponding to the 1, 5, 8, 14 positions are underlined. Sequences of three mutant forms of the region are shown; mutations are shown in bold. dfi1-WWQQ, *dfi1_W305,308Q_-TAP*; dfi1-RKAA, *dfi1_R309A,K310A_ -TAP*; dfi1-EERR, *dfi1_E302,312R_-TAP*. B, Western blot probed with Strep-Tactin showing *in vitro* binding of Dfi1p tail constructs to immobilized bovine calmodulin. WT, GST-Dfi1p tail-Strep; ctl, GST-Strep; WWQQ, GST-Dfi1-WWQQ-Strep; RKAA, GST-Dfi1-RKAA-Strep; EERR, GST-Dfi1-EERR-Strep. Fractions were I, input; F, flow through; W, wash; E, elution. Equal amounts of protein were either loaded as input or incubated with immobilized calmodulin. Numbers below blot indicate the amount of protein in each lane as a percentage of the total protein that was recovered from the column. All samples were run on the same gel; the order of the WT lanes was changed for clarity.

Many different calmodulin binding motifs have been characterized. One of the main types of calcium-dependent calmodulin binding motifs, found in calcineurin and many other Ca^2+^/calmodulin-binding proteins, is the 1-5-8-14 motif, characterized by hydrophobic residues at amino acids 1, 5, 8 and 14 and several basic residues conferring a net positive charge [18]. As shown in Fig. 1A, the C-terminal, cytoplasmic tail of Dfi1p contains a putative 1-5-8-14 calmodulin binding motif. To determine whether this region of Dfi1p binds to calmodulin, the 44-amino acid C-terminal tail of Dfi1p was dually tagged with glutathione S-transferase (GST) and *Strep*-tag (strep), translated *in vitro* and incubated with immobilized bovine calmodulin in the presence of calcium. Bovine calmodulin has 72% protein sequence identity with *C. albicans* calmodulin [19]. Proteins bound to the calmodulin beads were eluted using the calcium chelator EGTA to release proteins that bound to calmodulin in a calcium-dependent manner and detected by Western blotting. When GST-Dfi1 tail-Strep was incubated with calmodulin, the elution fraction contained 25% of the total protein that was recovered from the column (Fig. 1B, WT). When a construct containing a linker region in place of the Dfi1p tail was used, no protein was detected in the elution fraction, indicating that the Dfi1p tail, not the protein tags, bound to calmodulin *in vitro* (Fig. 1B, ctl).

### Mutations in Dfi1p affect calmodulin binding

To demonstrate that the putative calmodulin binding motif of Dfi1p was the region responsible for binding to calmodulin *in vitro*, three different mutants were constructed. The *dfi1_R309A,K310A_* mutation (dfi1-RKAA) changes the charge of the region to be further from the consensus sequence, from a net +1 to a net -1; the *dfi1_E302,312R_* mutation (dfi1-EERR) increases the net positive charge from +1 to +5, and the *dfi1_W305,308Q_* mutation (dfi1-WWQQ) substitutes two critical hydrophobic residues at positions 5 and 8 within the 1-5-8-14 motif (Fig. 1A). Based on the effects of similar mutations in the V2 vasopressin receptor [20] and sphingosine kinase 1 [21], the *dfi1-RKAA* and *dfi1-WWQQ* mutations were predicted to disrupt calmodulin binding, whereas the *dfi1-EERR* mutation should retain calmodulin binding activity. Mutant forms of the Dfi1 tail tagged with GST and Strep were translated *in vitro* and incubated with immobilized bovine calmodulin in the presence of calcium as before. The dfi1-RKAA and dfi1-WWQQ mutant proteins exhibited markedly reduced binding to calmodulin (Fig. 1B, 3% and 0% of the protein was eluted with EGTA) whereas the dfi1-EERR mutant protein retained the ability to bind calmodulin (30% of the protein was eluted with EGTA, Fig. 1B). Therefore, the predicted calmodulin binding motif of Dfi1p was important for binding to calmodulin *in vitro*; the features that define the motif must be intact in order for the Dfi1p tail to bind to calmodulin.

### Calmodulin binding motif of Dfi1p is important for invasion of agar medium

**Figure 2:**
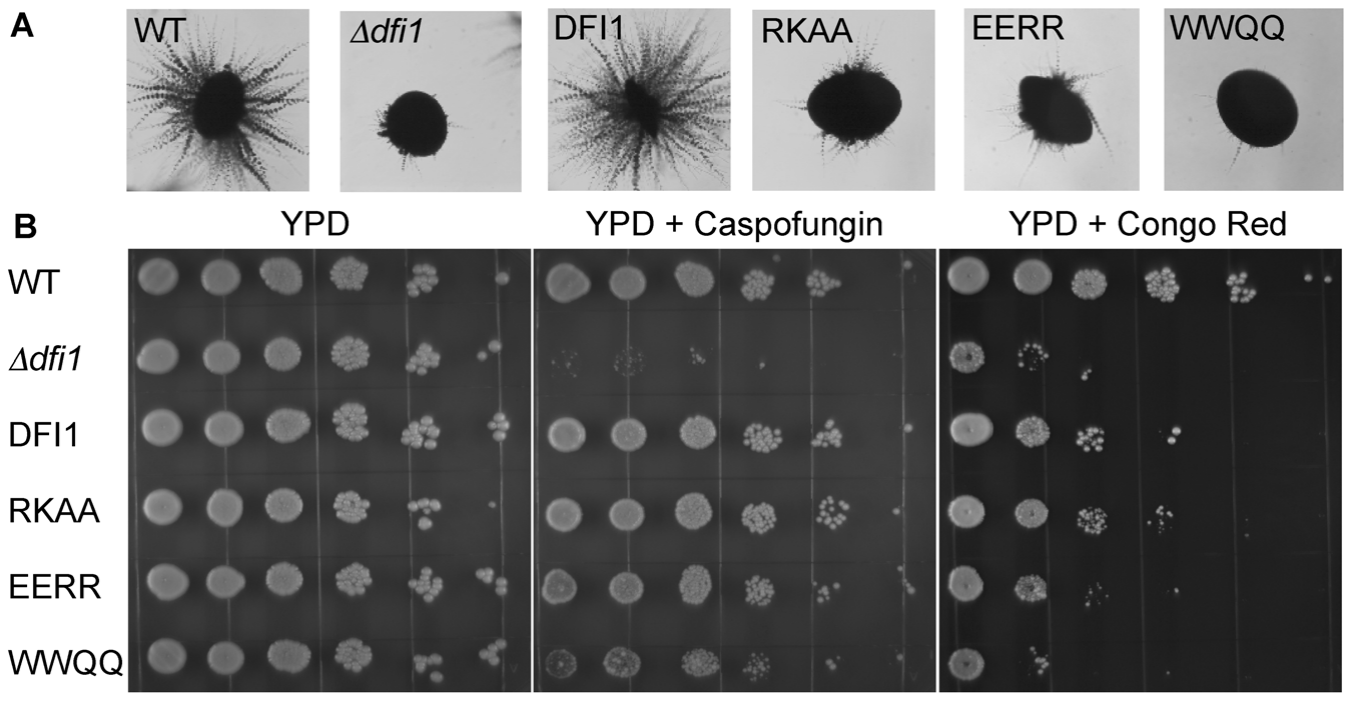
Effects of mutations in the calmodulin binding region of Dfi1p on filamentation and drug susceptibility. Panel A, *C. albicans* cells were grown embedded in an agar matrix. Colonies growing within the agar were visualized at 4× magnification; representative colonies are shown. Panel B, Exponentially growing *C. albicans* cells were serially diluted and plated on YPD with or without caspofungin (90 ng/mL) or congo red (200 µg/mL). WT, wild type; *Δdfi1*, *Δdfi1* null; TAP, *DFI1-TAP*; RKAA, *dfi1-RKAA-TAP*; EERR, *dfi1-EERR-TAP*; WWQQ, *dfi1-WWQQ-TAP*.

To determine whether the calmodulin binding domain of Dfi1p was important for the function of Dfi1p, the *dfi1-RKAA*, *dfi1-EERR*, and *dfi1-WWQQ* point mutations were introduced into the full-length *DFI1* gene with a 3′ epitope tag (TAP) and the mutant constructs were integrated into the *DFI1* locus of *C. albicans*. These mutant strains were then grown on the surface of or embedded within YPS 1% agar medium and incubated at 25°C. Under these conditions, wild type *C. albicans* filamented and invaded the agar, whereas *Δdfi1* null mutants did not (Figure 2A and reference [12]). The *dfi1-WWQQ-TAP* (2.2% filamentous colonies), *dfi1-RKAA-TAP* (2.5% filamentous colonies), and *dfi1-EERR-TAP* (1.9% filamentous colonies) mutants were defective in invading the agar in comparison with the wild type *DFI1-TAP* (89% filamentous colonies) (Fig. 2A). All strains grew at a rate similar to that of the wild type strain in liquid medium. In addition, these strains germinated and elongated hyphae similarly to the wild type strain in YPD liquid medium supplemented with serum (10%) or Spider liquid medium at 37°C (data not shown), demonstrating that the mutants were capable of forming filamentous hyphae when stimulated by different cues.

**Figure 3:**
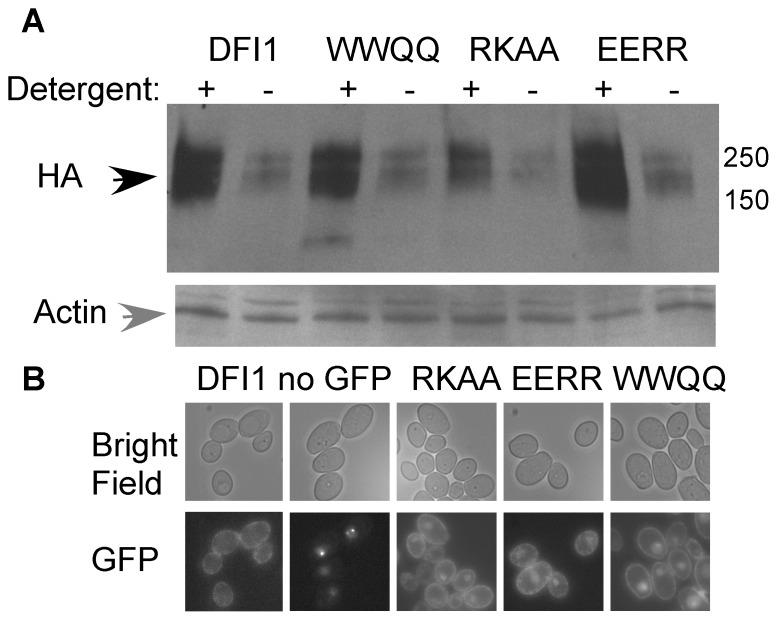
Expression and localization of Dfi1p mutant proteins. A, Wild type and mutant Dfi1-TAP protein are extractable with detergent. Cells were grown in YPD overnight, collected and total protein extracted in extraction buffer with (+) or without (−) 1% triton-X100 and 0.5% sodium deoxycholate. Equal amounts of total protein were fractionated on an SDS-PAGE gel and Western blotted with anti-HA (top) to detect Dfi1p (black arrow) or anti-actin (gray arrow) as a loading control (bottom). DFI1, *DFI1-TAP*; WWQQ, *dfi1-WWQQ-TAP*; RKAA, *dfi1-RKAA-TAP*; EERR, *dfi1-EERR-TAP*. Molecular weight markers are shown to the right (in kDa). B, Dfi1-GFP strains show GFP fluorescence at the periphery of the cell. Top, bright field; Bottom, GFP fluorescence. DFI1, *DFI1-GFP*; no GFP, *Δdfi1* null with no GFP; RKAA, *dfi1-RKAA-GFP*; EERR, *dfi1-EERR-GFP*; WWQQ, *dfi1-WWQQ-GFP*.

The Dfi1p protein was expressed at similar to WT levels in the mutant strains. The amount of extractable Dfi1p was slightly higher in the *dfi1-EERR-TAP* strain and slightly lower in the *dfi1-RKAA-TAP* strain as compared to the wild type *DFI1-TAP* strain (Fig. 3A), although these minor differences were not statistically significant (paired t-test; *dfi1-EERR-TAP* mean 2.2-fold *DFI1-TAP* expression, p = 0.07; *dfi1-RKAA-TAP* mean 0.74-fold *DFI1-TAP* expression, p = 0.40). Also, despite the presence of autofluorescent spots (clearly seen in the strain lacking GFP), expression of mutant and wild type *DFI1* tagged with GFP resulted in fluorescence at the periphery of the cells (Fig. 3B), demonstrating that the mutant proteins, like the wild type protein, were localized to the cell surface. Therefore, the normal production and localization of mutant proteins showed that the calmodulin binding motif of Dfi1p was important for invasive filamentation in response to growth in contact with agar.

### Growth on cell wall targeting agents

In the absence of Dfi1p, strains are hyper-susceptible to cell wall targeting agents caspofungin and Congo red [12]. To determine whether these mutations alter growth in the presence of these compounds, the mutant strains were grown to late exponential phase in YPD and plated on YPD or YPD supplemented with either 90 ng/mL caspofungin or 200 µg/mL Congo red (Fig. 2B). All strains yielded colonies when plated on YPD only (Fig. 2B) but the *Δdfi1* null exhibited low plating efficiency (reduced numbers of colonies for a given number of cells) on media containing caspofungin or Congo red. The *dfi1-RKAA-TAP* and *dfi1-EERR-TAP* mutants produced colonies on media containing caspofungin or Congo red, like the wild type *DFI1-TAP* strain. However, the *dfi1-WWQQ-TAP* mutant was more susceptible to these agents. Interestingly, the colonies in all of the mutant strains were smaller on media containing caspofungin or Congo red than on YPD alone, suggesting a slight growth defect on these agents even though the plating efficiencies for the *dfi1-RKAA-TAP* and *dfi1-EERR-TAP* mutants were similar to the wild type *Dfi1-TAP* strain. Therefore, mutations that change the charge of the calmodulin binding motif did not affect growth on cell wall targeting agents, but mutational change of the hydrophobic residues resulted in sensitivity to the agents. Interestingly, the *dfi1-WWQQ-TAP* mutant was more sensitive to Congo red than to caspofungin, indicating a difference in the effect of these two agents.

### Mutations in the calmodulin binding motif of Dfi1p compromise Cek1p activation during growth in contact with agar medium

**Figure 4:**
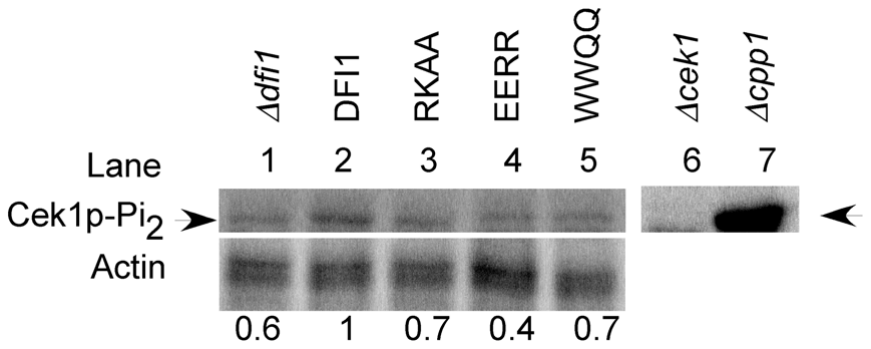
Mutations in Dfi1p compromise signaling to Cek1p in response to growth agar. Colonies were grown on YPS 1% agar for 4 days at 25°C. Western blots were run to detect dually phoshporylated Cek1p (top) and actin (bottom). Numbers indicate the amount of activated Cek1p (arrow) normalized to actin and shown relative to the *DFI1-TAP* strain grown on agar; lanes 1–5 are shown relative to lane 2. The experiment was repeated 5 times and representative blots are shown. Control lanes show that in the *cek1* null mutant, phosphoCek1p was not detected (lane 6) and in the *cpp1* null mutant, higher levels of phosphoCek1p were detected (lane 7). Δdfi1, *Δdfi1* null; DFI1, *DFI1-TAP*; RKAA, *dfi1-RKAA-TAP*; EERR, *dfi1-EERR-TAP*; WWQQ, *dfi1WWQQ-TAP*; Δ*cek1*, Δ*cek1* null; Δ*cpp1*, Δ*cpp1* null.

The MAP kinase Cek1p is activated when cells are grown in contact with agar medium and Dfi1p is required for full activation under these conditions [12]. To determine whether mutation of the calmodulin binding motif would affect the activation of Cek1p, the mutant strains were grown on YPS 1% agar at 25°C for four days. Cells were scraped off of the agar and extracted. Activated Cek1p was detected by Western blotting with antibody that recognizes the dually-phosphorylated form of p42/44 MAP Kinases; the Cek1p band was identified by its apparent molecular weight, absence in the *Δcek1* null mutant strain and hyperphosphorylation in the *Δcpp1* null mutant strain, as observed previously [12]. Levels of activated Cek1p were normalized to actin and compared to levels in strains carrying the wild type *DFI1-TAP* allele. When grown on the surface of agar medium, the *Δdfi1* null mutant strain (Fig. 4, lane 1) showed low levels of activated Cek1p in comparison with the strain carrying WT *DFI1-TAP* (Fig. 4 lane 2). In contrast, levels of phospho-Cek1p are undetectable during growth in liquid medium, as shown previously [12], [22]. Similarly, the *dfi1-RKAA-TAP* (p = 0.009, paired t-test; Fig. 4, lane 3) and *dfi1-WWQQ-TAP* (p = 0.005, paired t-test; Fig. 4, lane 5) mutant strains exhibited lower levels of activated Cek1p than the wild type *DFI1-TAP* strain. The *dfi1-EERR-TAP* (p = 0.063, paired t-test; Fig. 4, lane 4) mutant strain showed a trend towards lower levels of activated Cek1p, although this result was not statistically significant. Therefore, mutations that reduced calmodulin binding reduced Cek1p activation during growth in contact with agar.

### Increasing intracellular calcium results in Dfi1p-dependent Cek1p activation

**Figure 5:**
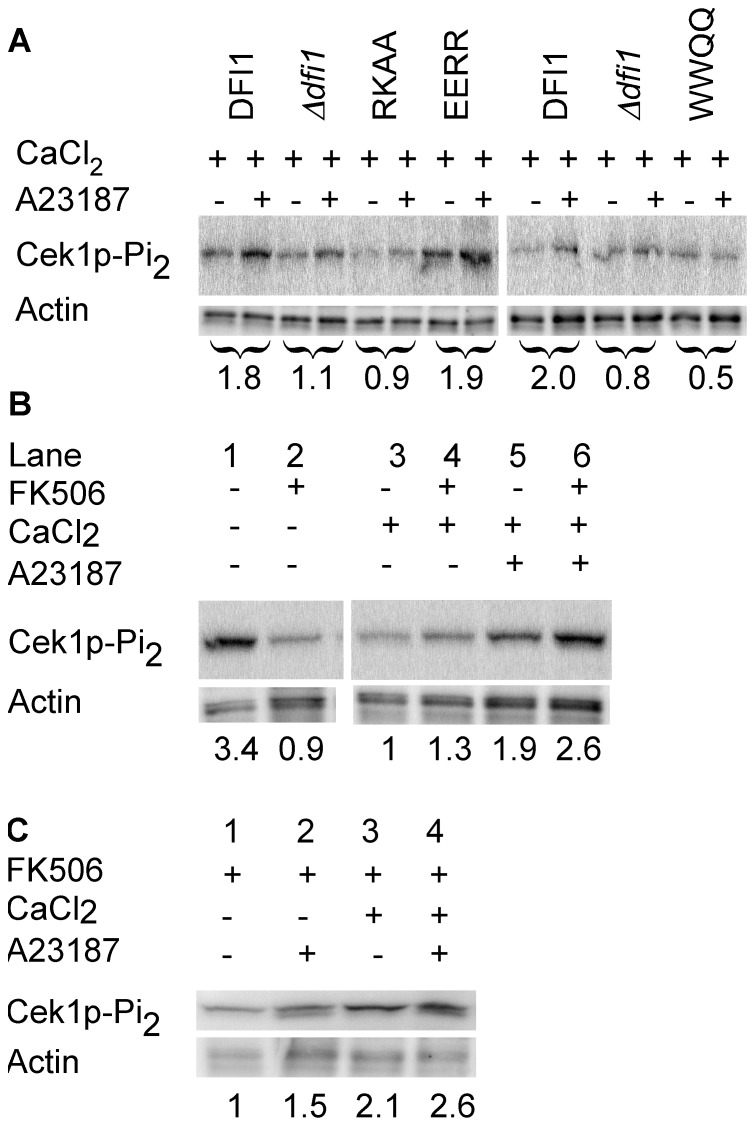
Activation of Cek1p in the presence of calcium ionophore A23187 and calcium. *C. albicans* cells growing exponentially were treated as described below for 30 minutes. Numbers below the blots in Panel A show the fold change in dually phosphorylated Cek1p in the presence of A23187 relative to the vehicle control for each strain. Lower numbers in Panels B and C indicate relative amount of dually phosphorylated Cek1p normalized to actin and shown relative to lane 3 (panel B), or cells treated with FK506 only in the absence of calcium (panel C, lane 1). Experiments were repeated 3 times; representative blots are shown. Panel A, Mutations in the calmodulin binding motif of Dfi1p affect A23187-dependent Cek1p activation. Dfi1p complemented and mutant strains were treated with 4 µM A23187 (+) or 100% ethanol (−) as a vehicle control. DFI1, *DFI1-TAP*; *dfi1*, *Δdfi1* null; RK, *dfi1-RKAA-TAP*; ER, *dfi1-EERR-TAP*; WQ, *dfi1-WWQQ- TAP*. Panels B and C, Activation of Cek1p is calcium dependent and independent of calcineurin. Wild type cells were grown in the presence of 1 mM CaCl_2_, 2 µg/mL FK506, 4 µM A23187 and/or the appropriate vehicle controls. Panel B, all lanes were from the same gel, and irrelevant lanes were removed.

To show that interaction between Dfi1p and calmodulin promotes Dfi1p signaling in the cell, we asked whether an increase in intracellular calcium would lead to the activation of Cek1p in a Dfi1p-dependent manner in the absence of contact with agar medium. To increase intracellular calcium, the calcium ionophore A23187 was used and Dfi1p-dependent Cek1p activation was used as a read-out for Dfi1p signaling. In wild type cells, treatment with A23187 resulted in a 1.8-fold increase in Cek1p activation that was not observed in *Δdfi1* null mutant cells (p = 0.0078, paired t-test; Fig. 5A). Therefore, treatment of cells with A23187 in the presence of calcium resulted in Dfi1p-dependent Cek1p activation.

### Increased intracellular calcium does not lead to Cek1p activation if Dfi1p is defective in calmodulin binding

To demonstrate that calmodulin binding to Dfi1p is required for Cek1p activation in response to increased intracellular calcium, *dfi1* mutants altered in calmodulin interaction were studied. The experiment was conducted as in Fig. 5A, using *dfi1-RKAA-TAP*, *dfi1-EERR-TAP* or *dfi1-WWQQ-TAP* mutant strains as well as WT *DFI1-TAP* and the *Δdfi1* null mutant strain. As shown in Fig. 5A, Cek1p activation did not increase in the *dfi1-RKAA-TAP* and *dfi1-WWQQ-TAP* strains upon treatment with A23187 in the presence of calcium. Therefore, calmodulin binding to Dfi1p is required for Cek1p activation in response to increased intracellular calcium concentration.

The *dfi1-EERR-TAP* mutant retains the ability to bind calmodulin. In this mutant strain, Cek1p activation increased upon A23187 exposure in the presence of calcium. Therefore, Dfi1p dependent activation of Cek1p in response to increased intracellular calcium requires the ability of Dfi1p to bind calmodulin.

### Dfi1p-dependent Cek1p activation requires calcium

To demonstrate that the effect of A23187 on Cek1p activation requires calcium, cells were grown in media without calcium. This growth condition resulted in Cek1p activation in the absence of A23187 (Fig. 5B, lane 1), which was reduced by the addition of calcium (Fig. 5B, lane 3). However, previous studies showed that activation of Cek1p in the absence of calcium is dependent on calcineurin and can be inhibited by treatment of cells with FK506, a calcineurin inhibitor [23]. Therefore, wild type cells grown without calcium were incubated with FK506 or 100% ethanol as a vehicle control. As shown in Fig. 5B lane 2, inhibiting calcineurin with FK506 reduced background levels of activated Cek1p in medium lacking calcium. Therefore, we inhibited calcineurin with FK506 in order to test the importance of calcium for Cek1p activation in response to A23187 treatment. Cells were grown in the absence or presence of calcium and treated with FK506 together with either A23187 or 100% ethanol (Fig. 5C). Under these conditions, cells grown without calcium and without A23187 had low levels of Cek1p activation (Fig. 5C, lane 1). Cells treated with calcium and A23187 in the presence of FK506 showed increased levels of Cek1p activation (Fig. 5C, lane 4). Importantly, cells grown in the absence of calcium and treated with A23187 and FK506 showed lower levels of Cek1p activation (Fig. 5C, lane 2). Therefore, under these conditions, A23187 did not stimulate high levels of Cek1p activation unless extracellular calcium was present. Some increase in activation of Cek1p was observed with the addition of calcium alone (Fig. 5C, lane 3). These results also showed that FK506 did not inhibit Cek1p activation in response to treatment with A23187 in the presence of calcium (Fig. 5B, lanes 5, 6). Thus, these data demonstrate that, in the presence of FK506, activation of Cek1p by A23187 treatment is dependent on calcium and not dependent on calcineurin.

### Importance of Dfi1p-dependent signaling for virulence in a mouse model of *C. albicans* systemic infection

**Figure 6:**
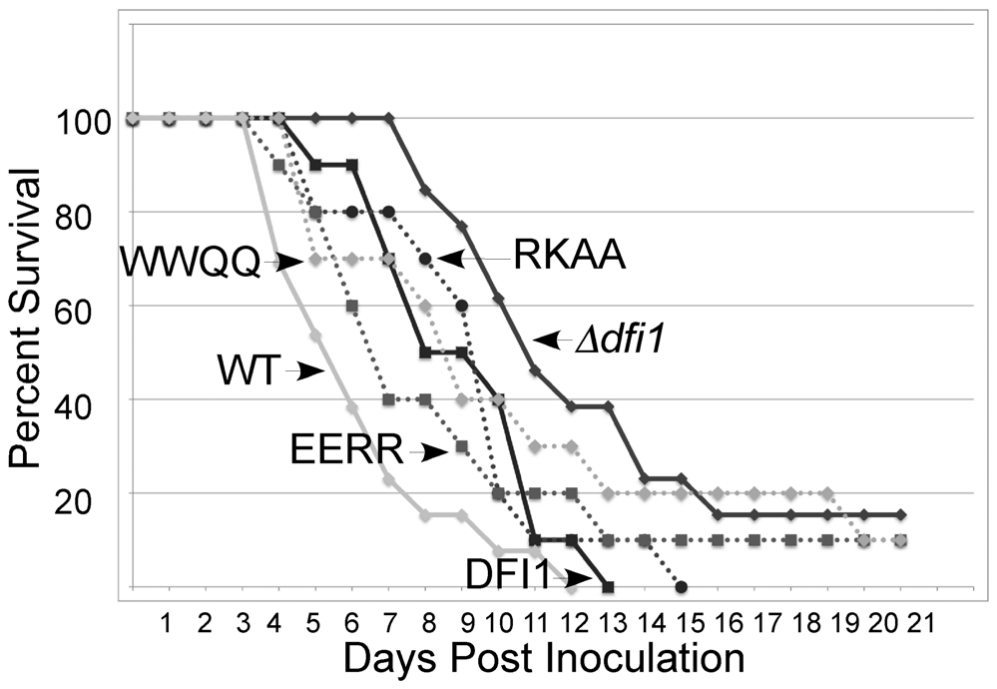
Calmodulin-dependent signaling is not required for virulence. CF-1 mice were inoculated with 3×10^5^ *C. albicans* cells and monitored for survival. Animals were euthanized when moribund. WT; DFI1, *DFI1-TAP*; *dfi1*, *Δdfi1* null; RKAA, *dfi1-RKAA-TAP*; EERR, *dfi1-EERR-TAP*; WWQQ, *dfi1-WWQQ- TAP*.

To determine whether Dfi1p-dependent signaling was important for *C. albicans* virulence, the murine model of disseminated candidiasis was used. Previous results demonstrated that deletion of *DFI1* attenuates the virulence of *C. albicans* [12]. To test the importance of calmodulin-dependent signaling, we studied the virulence of the *dfi1* point mutants. To test the effects of these mutations on *C. albicans* virulence, the wild type strain, the *Δdfi1* null mutant, the complemented *DFI1-TAP* strain, and the three point mutants were inoculated intravenously into mice and survival was monitored. The wild type strain and the *DFI1-TAP* complemented strain caused lethal infections and all mice succumbed (Fig. 6). Consistent with previous results [12], the *Δdfi1* null strain was attenuated in virulence (p = 0.0239, log rank test vs. the *DFI1-TAP* complemented strain; Fig. 6). Mutants that were altered in the calmodulin binding motif were indistinguishable from the *DFI1-TAP* strain, indicating that mutation of this region does not compromise virulence (Fig. 6). Therefore, calmodulin-dependent signaling is not required for lethal infection in this model.

## Discussion

Calmodulin is a ubiquitous and well-characterized eukaryotic protein with many roles in mammals and fungi [13], [14], [16], [24], [25]. Previous work has shown that calcineurin, which is activated by calmodulin, plays a role in drug tolerance and regulating the cell wall, and there is cross-talk between the calcineurin and Mck1p MAPK pathways in mammals, *S. cerevisiae*, and *S. pombe* [26], [27], [28]. However, this work represents the first time calmodulin has been shown to bind to a plasma membrane protein in *C. albicans*. To the authors' knowledge, this is the first report that calcium calmodulin signaling leads to Cek1p activation independently of calcineurin.

We propose that when cells are grown in contact with an agar matrix, changes in the cell wall or plasma membrane are sensed by Dfi1p, which alters its conformation, binds to calmodulin and relays a signal that activates Cek1p. *dfi1* mutants that are defective in interacting with calmodulin are defective in activating Cek1p to wild type levels in response to contact with an agar matrix.

Dfi1p may change conformation upon calmodulin binding. Other proteins such as the Epidermal Growth Factor Receptor (EGFR) and SNARE proteins are known to function in this way [25], [30]. The mammalian v-SNARE VAMP2, yeast v-SNARE Nyv1p, and Dfi1p share similar 1-5-8-14 calmodulin binding motifs near their transmembrane domains [29]. In VAMP2, this region binds to calmodulin or to membrane lipids in a mutually exclusive manner that is important for membrane fusion and exocytosis [29]. Similarly, the membrane-juxtaposed region of Dfi1p may bind alternatively to calmodulin and another partner, such as another protein or lipids in the plasma membrane. Binding to calmodulin may allow Dfi1p to respond to an extracellular signal, generated by growth on an agar matrix, and relay the signal through the cell to phosphorylate the MAP kinase Cek1p.

Our results suggest that calmodulin binding to Dfi1p leads to Cek1p activation in the presence of A23187 and calcium. Disrupting the calmodulin binding motif affects Cek1p activation in the presence of A23187. In addition, mutations in the calmodulin binding motif of Dfi1p render the protein unable to support filamentous invasion. Mutation of the calmodulin binding motif of Dfi1p so that the net charge changed from +1 to +5 resulted in defective filamentous invasion. The +1 net charge of the Dfi1p calmodulin binding motif is lower than the charge of many calmodulin binding motifs and may facilitate quick release of calmodulin. Therefore, the defect in filamentous invasion and Cek1p activation of the *dfi1-EERR-TAP* mutant suggests that the interaction between Dfi1p and calmodulin is transient; Dfi1p must both bind to and release calmodulin in order to change conformation and signal to Cek1p to support filamentation. Interestingly, in liquid conditions when intracellular calcium is increased, the dfi1-EERR-TAP mutant protein supports Cek1p activation. This result suggests that the dfi1-EERR-TAP mutant protein is capable of signaling to Cek1p but does not respond normally during growth in contact with an agar surface. During growth in contact with agar, the number of activated Dfi1p molecules may be low and efficient binding and release of calmodulin may be needed. Alternatively, the *dfi1-EERR-TAP* mutation may affect the binding of other Dfi1p binding partners that affect the ability of the strain to filament during growth in agar.

In the mouse model of disseminated candidiasis, the calmodulin-binding mutants are virulent. Therefore, Dfi1p may be able to become activated via more than one mechanism in the animal. Interestingly, a different mutant, *dfi1_G273,277L_-TAP*, that has been shown previously to be defective in invasive filamentation and Cek1p activation in response to contact with an agar matrix [12], is attenuated for virulence (T.R.D. and C.A.K., unpublished observations), arguing that some Dfi1p functions are important for pathogenicity. Thus, signaling pathways are more complex in the host than in laboratory growth and although calmodulin signaling is important for Dfi1p function in laboratory conditions, redundant mechanisms may allow Dfi1p to function without calmodulin binding during host infection.

Many pathways regulate filamentation in *Candida albicans*. Likewise, *C. albicans* virulence is controlled by numerous pathways. Redundancy in the mechanisms that promote virulence is a common theme in *C. albicans* biology and probably contributes to the success of the organism as a pathogen.

## Materials and Methods

### Strains and growth conditions

All strains are listed in Table S1. *C. albicans* cells were routinely grown in YPD (1% Yeast extract, 2% peptone, 2% glucose), YPS (1% Yeast extract, 2% peptone, 2% sucrose) or CM-U (complete medium minus uridine, [30]) at 25 or 30°C. The *cek1* and *cpp1* null mutants were kindly provided by M. Whiteway [9], [31]. BWP17 was kindly provided by A. Mitchell. *E. coli* DH5α or XLIBlue strains grown in L broth plus the appropriate antibiotics were used to propagate plasmids.

### Construction of Dfi1p mutant strains

*C. albicans* strains were derived from strain BWP17 [32]. The *dfi1* null, and DFI1-TAP strains have been described previously [12]. The TAP tag is a tandem His6x and HA tag. Mutant *dfi1* alleles were made by overlap PCR with mutagenic primers using pNEBDFI1-TAP (*DFI1-TAP* cloned into the pNEB193 vector, New England Biolabs, [12]) or pSPLDFI1-GFP (*DFI1-GFP* cloned into the SAT placer, [12]) as a template. All primers used in this study are listed in Table S2. The first round primer pairs were TD47/TD28 and TD48/PZ134 (*dfi1-WWQQ-TAP*), TD49/TD28 and TD50/PZ134 (*dfi1-RKAA-TAP*), and TD51/TD28 and TD52/PZ134 (*dfi1-EERR-TAP*). The second round primer pair was TD28/PZ134 for all strains. The fragments were digested with Xho1 and Mlu1 and ligated into pSPLDFI1-TAP or pSPLDFI1-GFP [12]. Plasmids were linearized with BglII and transformed into a *dfi1* null strain.

All PCR reactions used Hi-Fi polymerase (Invitrogen) or Taq polymerase (Phoenix Lab, Tufts Medical Center) and were confirmed by sequencing. All restriction enzymes and ligase were purchased from New England Biolabs.

### Constructs containing Dfi1p C-terminal tails

pGEX-GST-ctDFI1 was made by annealing primers TD22/TD23, TOPO cloning into vector pCR2.1 (Invitrogen), digesting with BamHI and NotI and ligating into the pGEX5.1 plasmid. Mutant *dfi1* alleles were made by overlap PCR with mutagenic primers using pGEX-GST-ctDFI1 as a template. The first round primer pairs were TD39/PZ374 and TD40/PZ373 (pGEX-GST-*dfi1-WWQQ*), TD41/PZ374 and TD42/PZ373 (pGEX-GST-*dfi1-RKAA*), and TD63/TD60 and TD61/TD62 (pGEX-GST-*dfi1-EERR*). The second round primer pair was PZ373/PZ374 (pGEX-GST-*dfi1-RKAA*, GST-*dfi1-WWQQ*) or TD62/TD63 (pGEX-GST-*dfi1-EERR*).

### *In vitro* calmodulin binding assay

Two-round PCR protocols (Qiagen) were used to generate DNA fragments for *in vitro* translation reactions. pGEX-GST-ctDFI1 constructs were amplified using primers PZ326 and either PZ352 (GST-STREP control) or PZ330 (GST-ctDFI1-STREP and all mutants). All primers contained linker sequences needed for the second round of PCR, which was performed following the manufacturer's recommendations and introduced a c-terminal STREP tag to the constructs (Qiagen EasyXpress Linear Template Kit). PCR reactions were gel purified using Qiaex II resin and 250 ng DNA used in each translation reaction (PURExpress *In vitro* Protein Synthesis Kit, New England Biolabs E6800S). Translation reactions were performed with 20 U of murine RNAse inhibitor (NEB M0314S) according to the manufacturer's instructions. Protein production was confirmed by Western blot with Strep-Tactin-HRP (IBA GmbH).

For the binding assay, calmodulin affinity resin (Stratagene 214303-52) was washed three times with 5 bed volumes wash buffer (PBS, 2 mM CaCl_2_, 0.05% Tween 20), centrifuging for 2 minutes at 500 xg between washes. Beads were then resuspended in wash buffer to make a 50% slurry. *In vitro* translated protein (10 µL) was mixed with 40 µL wash buffer, added to 80 µL of the 50% slurry and allowed to rotate at 4°C for 4 hours. The incubation was then transferred to Micro Bio-Spin Chromatography Columns (Bio-Rad 732-6204) and spun at 500 xg for 30 seconds to collect flow through. Resin was washed twice by adding 100 µL wash buffer to the column, letting the resin sit at room temperature for 1 minute, and spinning at 500 xg for 30 seconds. Protein was eluted by adding elution buffer (PBS, 0.05% Tween, 4 mM EGTA) to the column, incubating at room temperature for 1 minute and spinning at 500 xg for 30 seconds. Each fraction was boiled in protein loading buffer (60 mM tris pH 6.8, 2% SDS, 2% glycerol, 0.005% bromophenol blue, 300 mM β-mercaptoethanol, 10 µM dithiothreitol), and half of each fraction was loaded on an SDS-PAGE. An equivalent amount of *in vitro* translation reaction for the input was also loaded. After electrophoresis, the proteins were transferred onto 0.2 µm PVDF, blocked in PBS 0.1% Tween 3% BSA and probed with Strep-Tactin-HRP (IBA GmbH). The signal was produced using Pierce ECL Western Blotting Substrate (32209) and detected on Kodak X-OMAT Blue XB film. Experiments were performed at least 3 times per construct, and a representative experiment is shown. Quantification was performed using a Gel Logic 100 Imaging System with the program Kodak 1D, version 3.6.

### Invasion of agar medium

*C. albicans* strains were grown to early exponential phase and mixed with molten agar (YPS 1% agar) as described previously [12]. Plates were incubated for 4 days at 25°C, at which point 100% of colonies from WT strains were filamentous, and colonies visualized at 4× magnification. Colonies were scored as filamentous if 20 or more filaments protruded from the colony. Three independent isolates of each strain were tested in triplicate; representative colonies are shown.

### Growth in the presence of cell wall targeting agents

*Candida albicans* strains grown at 30°C in YPD were diluted and allowed to grow to OD600  =  approximately 1.5. Cultures were serially diluted and 5 µL of each dilution spotted on YPD with or without caspofungin (90 ng/mL) or Congo red (200 µg/mL). Plates were incubated at 30°C for 72 hours. Three independent isolates of each strain were tested in triplicate.

### Protein extraction with detergent

*C. albicans* cells were grown in YPD overnight at 30°C, washed with PBS and resuspended in lysis buffer (50 mM Tris pH 7.5, 100 mM NaCl, 0.5 mM EDGA) with or without 1% Triton-X100 and 0.5% Na deoxycholate with 0.5 mm zirconia silica beads. Cells were broken on a Turbomix vortex attachment (Fisher) with 6 cycles of 30 seconds on the vortex and 1 minute on ice. Protein concentration was determined using a Pierce micro BCA protein concentration kit (Pierce 23235) and 80 µg (Dfi1-HA) or 20 µg (actin) was run on a 4–15% SDS-PAGE gel (Bio-rad), transferred onto 0.2 µm PVDF and Western blotted with mouse anti-HA (Covance 16B12, 1∶1000 overnight incubated at 4°C) or rabbit anti-actin (Sigma A5060, 1∶10000 overnight incubated at 4°C). Goat-anti-mouse-HRP (Bio-rad 170-6516) or Goat-anti-rabbit-HRP (Invitrogen 656120) was used as a secondary antibody and the signal was produced using Pierce ECL Western Blotting Substrate (32209) and detected on Kodak X-OMAT Blue XB film. Experiments were performed 3 times, and a representative experiment is shown. Quantification was performed using a Gel Logic 100 Imaging System with the program Kodak 1D, version 3.6.

### GFP localization

Exponentially growing *C. albicans* strains were washed with PBS, mounted on glass slides and visualized with Openlab (Improvision, version 5.5.1) on a Zeiss Axiovert 200 M microscope with a 40x lens and standard FITC fluorescent filter cube (Chroma Technology Corp.). Pictures were taken with a camera (C4742-95-12ERG; Hamamatsu Photonics) controlled by Openlab version 5.5.1 (PerkinElmer).

### Cek1p activation in response to contact with agar medium

Cells were grown and protein extracted as previously described [12]. Briefly, exponentially growing *Candida albicans* cells were plated for single colonies on YPS plates with 1% agar and incubated at 25°C for 4 days. Colonies were washed off the plates with cold PBS and collected over ice. Total protein was extracted in RIPA buffer supplemented with phosphatase and protease inhibitors (50 mM tris pH 8, 150 mM NaCl, 0.1% SDS, 1% NP40, 0.5% Na deoxycholate, 20 mM NaF, 10 mM Na orthovanadate, 50 mM β-glycerol phosphate, 50 mM Na pyrophosphate, 2 mM PMSF, 10 µL/mL fungal specific protease inhbitor (Sigma P8215), 1 Complete tablet/10 mL (Roche 04693116001)) using 0.5 mm zirconia silica beads on a Turbomix vortex attachment (Fisher) with 6 cycles of 30 seconds on the vortex and 1 minute on ice. Protein concentration was determined using a Pierce micro BCA protein concentration kit (Pierce 23235) and 120 µg (Cek1p) or 20 µg (actin) total protein loaded on an 8.5% (Cek1p) or 10% (actin) SDS-PAGE and transferred onto 0.2 µm PVDF. Blots were blocked with 5% milk in TBS 0.05% tween and probed with anti-p42/44 (Cek1p-Pi_2_, Cell Signaling 4370, 1∶1000, overnight incubated at 4°C) or anti-actin (Sigma A5060). HRP-conjugated goat anti-rabbit (Invitrogen 656120) was used as a secondary antibody. Amersham ECL Plus Western Blotting Detection System (GE Healthcare RPN2132) was used to produce the signal, which was detected on a Syngene G:Box Chemi-XT4 GENE*Sys* imager. Blots were quantified using GeneTools (SynGene, version 4.02) using a standard curve run on each gel. This experiment was replicated 3 times and a representative blot is shown. Signals obtained with WT or *dfi1* mutant strains were compared using a paired t-test (Graphpad).

### Treatment with calcium ionophore A23187

*C. albicans* strains were grown for 8 hours at 30°C in complete minimal media without uridine (CM-U, [30]). For some experiments, CM-U was made with yeast nitrogen base lacking divalent cations and potassium phosphate (Sunrise Science Products 1540–250) and supplemented with 1 g/L potassium phosphate and 100 µM MgSO_4_. Cultures were then diluted 1∶1000 into 80 mL of the same fresh media and grown overnight. When the cultures reached approximately OD600 = 1, they were treated with 4 µM A23187 or an equivalent volume of 100% ethanol as a vehicle control for 30 minutes. Cultures were treated with 2 µg/mL FK506 or an equivalent volume of ethanol as a vehicle control and 10 mM CaCl_2_ or an equivalent volume of water as a vehicle control where indicated. Cells were collected over ice, washed with cold PBS and total protein extracted as described above. Equal amounts of protein (60 µg for Cek1p-Pi_2_ or 20 µg for actin) were loaded on an SDS-PAGE gel and Western blotted as described above. Experiments were performed 3 times per strain and condition; representative blots are shown.

### Mouse model of disseminated candidiasisis

*C. albicans* cells (WT, pcz24; *DFI1-TAP*, trd7; *Δdfi1* null (pcz25); *dfi1-RKAA-TAP* trd9; *dfi1-EERR-TAP*, trd10; *dfi1-WWQQ- TAP*, trd8) were grown at 30°C in CM-U for 24 hours, then washed 3 times in PBS. Cells were then resuspended in PBS at 3×10^6^ cells per mL and 3×10^5^ cells injected into the tail vein of 10 mice per strain. Survival time (days) was recorded. Mice were euthanized when moribund. The protocol was approved by the Tufts University School of Medicine Institutional Animal Care and Use Committee (Animal Welfare Assurance Number A-3775-01). Statistics were performed using a log-rank test from a Kaplan-Meier plot by Robin Ruthazer at the Tufts Medical Center Biostatistics Research Center.

## Supporting Information

Table S1 ***C. albicans*** **strains used in this study.**
(DOC)

Table S2 **Primers Used in this Study.**
(DOC)
