# Supplementary material for: Calmodulin Binding to Dfi1p Promotes Invasiveness of Candida albicans
Source: PLoS One. 2013 Oct 14;8(10):e76239. doi: 10.1371/journal.pone.0076239 (PMC3796530; doi:10.1371/journal.pone.0076239)
Supplement: Table S1 — C. albicans strains used in this study. (DOC) [file pone.0076239.s001.doc]

Table S1. *C. albicans* strains used in this study

| Strain | Description | Genotype | Source |
| --- | --- | --- | --- |
| pcz1 | Wild type | BWP17, *ura3Δ::imm434/URA3* |  |
| pcz5 | *dfi1* null | BWP17, *dfi1Δ*::FRT/*dfi1Δ*::FRT, *ura3Δ::imm434/URA3* |  |
| pcz9 | *DFI1-TAP* | pcz5, *dfi1Δ*/*dfi1::DFI1-His6HA-*SAT placer |  |
| trd1 | *DFI1_W305,308Q_-TAP* | pcz5, *dfi1Δ*/*dfi1::dfi1_W305,308Q_-TAP-*SAT placer | This work |
| trd2 | *DFI1_R309A,K310A_-TAP* | pcz5, *dfi1Δ*/*dfi1::dfi1_R309A,K310A_-TAP-*SAT placer | This work |
| trd3 | *DFI1_E302,312R_-TAP* | pcz5, *dfi1Δ*/*dfi1::dfi1_E302,312R_-TAP-*SAT placer | This work |
| pcz12 | *DFI1-GFP* | pcz5, *dfi1Δ*/*dfi1::DFI1-GFP-*SAT placer |  |
| trd4 | *DFI1_W305,308Q_-GFP* | pcz5, *dfi1Δ*/*dfi1::dfi1_W305,308Q_-GFP-*SAT placer | This work |
| trd5 | *DFI1_R309A,K310A_-GFP* | pcz5, *dfi1Δ*/*dfi1::dfi1_R309A,K310A_-GFP-*SAT placer | This work |
| trd6 | *DFI1_E302,312R_-GFP* | pcz5, *dfi1Δ*/*dfi1::dfi1_E302,312R_-GFP-*SAT placer | This work |
| pcz24 | Wild type | pcz1, *his1Δ*/*HIS1^+^, arg4Δ*/*ARG4^+^* |  |
| pcz25 | *dfi1* null | pcz5, *his1Δ*/*HIS1^+^, arg4Δ*/*ARG4^+^* |  |
| trd7 | *DFI1-TAP* | pcz25, *dfi1Δ*/*dfi1::DFI1-His6HA-*SAT placer | This work |
| trd8 | *DFI1_W305,308Q_-TAP* | pcz25, *dfi1Δ*/*dfi1::dfi1_W305,308Q_-TAP-*SAT placer | This work |
| trd9 | *DFI1_R309A,K310A_-TAP* | pcz25, *dfi1Δ*/*dfi1::dfi1_R309A,K310A_-TAP-*SAT placer | This work |
| trd10 | *DFI1_E302,312R_-TAP* | pcz25, *dfi1Δ*/*dfi1::dfi1_E302,312R_-TAP-*SAT placer | This work |
| pcz27 | *DFI1_G273,277L_-TAP* | pcz25, *dfi1Δ*/*dfi1::dfi1_G273,277L_-TAP-*SAT placer | This work |
| CCC55 | *cek1* null | CAI4, *cek1Δ::hisG*/*cek1Δ::(hisG-URA3-hisG)* |  |
| CCC81 | *cpp1* null | CAI4, *cpp1Δ::hisG*/*cpp1Δ::(hisG-URA3-hisG)* |  |

1. Zucchi PC, Davis TR, Kumamoto CA (2010) A *Candida albicans* cell wall-linked protein promotes invasive filamentation into semi-solid medium. Mol Microbiol 76: 733-748.

2. Csank C, Makris C, Meloche S, Schroppel K, Rollinghoff M, et al. (1997) Derepressed hyphal growth and reduced virulence in a VH1 family-related protein phosphatase mutant of the human pathogen Candida albicans. Mol Biol Cell 8: 2539-2551.

3. Csank C, Schroppel K, Leberer E, Harcus D, Mohamed O, et al. (1998) Roles of the Candida albicans mitogen-activated protein kinase homolog, Cek1p, in hyphal development and systemic candidiasis. Infect Immun 66: 2713-2721.
