# Supplementary material for: Calmodulin Binding to Dfi1p Promotes Invasiveness of Candida albicans
Source: PLoS One. 2013 Oct 14;8(10):e76239. doi: 10.1371/journal.pone.0076239 (PMC3796530; doi:10.1371/journal.pone.0076239)
Supplement: Table S2 — Primers Used in this Study. (DOC) [file pone.0076239.s002.doc]

Table S2: Primers Used in this Study

Primer Sequence Source

| TD22 | GGATCCAAACGTAACAACCGTGACTACGAAGGTGGTTGGACCTTCTGGCGTAAAAACGAAAAACTGGGTTCTGACGAATTCTTCAACGG | This study | |
| --- | --- | --- | --- |
| TD23 | GCGGCCGCTTATTAGCAAGAGAAGTTAGAACCCTGGTTGATGTTACGGTCACGAACACCCAGTTCACCGTTGAAGAATTCGTCAGAACCC | This study | |
| TD28 | ATTTTGCTTAGAGGTATAAGATGTTAGACGC | This study | |
| TD39 | GTCAGACCTTCCAGCGTAAAAACG | This study | |
| TD40 | TTTTACGCTGGAAGGTCTGACCACC | This study | |
| TD41 | TTCTGGGCGGCAAACGAAAAACTGGG | This study | |
| TD42 | TTTTCGTTTGCCGCCCAGAAGGTCC | This study | |
| TD47 | GGACAGACTTTCCAGAGAAAGAATGAGAAATTGGG | This study | |
| TD48 | ATTCTTTCTCTGGAAAGTCTGTCCACCTTCATAATCACGG | This study | |
| TD49 | GGACTTTCTGGGCAGCGAATGAGAAATTGGG | This study | |
| TD50 | TCTCATTCGCTGCCCAGAAAGTCCATCCACC | This study | |
| TD51 | GATTATAGAGGTGGATGGACTTTCTGGAGAAAGAATAGGAAATTGGG | This study | |
| TD52 | TCCTATTCTTTCTCCAGAAAGTCCATCCACCTCTATAATCACGG | This study | |
| TD60 | GTTTTCGGTTTTTACGCCAGAAGGTCCAACCACCTCGGTAGTCACGG | This study | |
| TD61 | ACCGAGGTGGTTGGACCTTCTGGCGTAAAAACCGAAAACTGGG | This study | |
| TD62 | CCTTTCGTCTTCAAGAATTATACACTCCGCTATCGCTACGTGACTGGG | This study | |
| TD63 | GCGATGAAGGTGATAAATGGCGAAACAAAAAGTTTGAATTGGGTTTGG | This study | |
| PZ134 | TGACGAAAACAACTCAACAACAAACG | [1] | |
| PZ326 | AGAAGGAGATAAACAATGTCCCCTATACTAGGTTATTGG | This study | |
| PZ330 | GGATGAGACCAGGCAGAGCAAGAGAAGTTAGAACCCTGG | This study | |
| PZ352 | GGATGAGACCAGGCAGAGATCCCACGACCTTCGATCAG | This study | |
| PZ373 | GATGGTGATGTTAAATTAACACAGTCTATGG | | This study |
| PZ374 | CAAGAATTATACACTCCGCTATCGCTAC | | This study |

1. Zucchi PC, Davis TR, Kumamoto CA (2010) A *Candida albicans* cell wall-linked protein promotes invasive filamentation into semi-solid medium. Mol Microbiol 76: 733-748.
